# Perceived physical literacy instruments for sports club coaches: further differentiated validation

**DOI:** 10.3389/fpsyg.2025.1589408

**Published:** 2025-09-12

**Authors:** Jiangping Fu, Weiping Zhang, Bing Cao, Huiling Yu, Hua Li

**Affiliations:** ^1^Nantong Institute of Technology, Nantong, China; ^2^Sports School, Nantong Normal College, Nantong, China; ^3^Zhengde Polytechnic, Nanjing University of Aeronautics and Astronautics, Nanjing, China; ^4^Tianjin Normal University, Tianjin, China; ^5^Anqing Vocational and Technical College, Anqing, China; ^6^Sports School, Sichuan Technology and Business University, Sichuan, China

**Keywords:** factor structure, perceived physical literacy, instrument, coaches, reliability, validity

## Abstract

**Background:**

The concept of physical literacy (PL) is a theoretical basis for public health. China has proposed PL indicators in its 2030 Sports Power Strategy through systematic administrative measures. Sports club coaches are an important force in promoting public health, and their PL is worthy of attention. The objective is to assess the factor structure of the Perceived PL Instrument (PPLI) in Simplified Chinese for sports club coaches.

**Methods:**

The 18-item PPLI was selected because of its efficiency and effectiveness for physical education teachers. The research team translated the questionnaire (English–Simplified Chinese). The questionnaire was distributed to coaches of school sports clubs and social sports clubs. The factor structure was established by exploratory factor analysis and confirmatory factor analysis (CFA).

**Results:**

The factor structure was a 4-factor, 9-item scale with satisfactory validity. Through exploratory factor analysis, item loadings ranged from 0.70 to 0.75 (Cronbach’s α, 0.83–0.87). Through CFA, factor loadings ranged from 0.78 to 0.86.

**Conclusion:**

The PPLI can be used as a reliable and valid instrument to test the PL of sports club coaches.

## 1 Introduction

To address the global phenomenon of physical inactivity, Whitehead (1993) developed the concept of Physical Literacy (PL; Whitehead, 1993). Based on each individual’s specific circumstances, Whitehead believes that PL is described as emphasizing and taking responsibility for six aspects of lifelong physical activity (motivation, confidence, physical ability, knowledge, environment, and understanding; Whitehead, 1993; Whitehead, 2001). Over the past three decades, PL has been extended from its original philosophical concepts, such as monism and phenomenology (Whitehead, 2001), to a wide range of fields, such as public health (Whitehead, 2010), while being blended by different perspectives due to regional cultural differences (Whitehead, 2019). PL is very popular and has become an indicator of national policies at the macro level (Bailey et al., 2023), such as Canada (ParticipACTION, 2025), Australia (Macdonald, 2013), New Zealand (Coaching Ireland, 2024), the United States (Society of Health and Physical Educators America, n.d.), Portugal (Tan et al., 2017), and China (Cairney et al., 2019). At the micro level, PL has been equally well received (Cairney et al., 2019). Researchers agree that PL may be related to lifelong physical behavior, and the basic framework of individual PL (motivation, confidence, and communication) has been identified through correlational studies with physical activity (Whitehead, 2019), such as in emerging adult populations (Gandrieau et al., 2023). As a basis for collaborative work, people do not seem to fully agree that this is an improvement (Bailey et al., 2023). At the operational level, researchers have put in a lot of effort (Lundvall, 2015; Robinson and Randall, 2017), starting with the development of various validation instruments [PPLA-Q (Mota et al., 2021), PLAY (Caldwell et al., 2021), PL-C Quest (Barnett et al., 2022), CAEPL (Chen et al., 2020), PFL (Lodewyk, 2019), Pre-PLAY (Cairney et al., 2018), PPLI (Sum et al., 2016), and CAPL (Longmuir et al., 2015)], to justify structural modeling in relation to different categories of populations, including early childhood (Lodewyk, 2019; Cairney et al., 2018), children (Pan et al., 2021), adolescents (Mota et al., 2021), and adults (Gandrieau et al., 2023). In recent years, research on the association of PL with quality of life (Yan et al., 2023) and self-efficacy (Sum et al., 2018) has been gradually carried out. Little is known about the assessment, implementation, and application of PL (Singh and Carl, 2023). The focus of our study explores the social configuration of PL in the public health perspective from the overall framework of PL.

From an individual health perspective, a variety of factors, such as regional, environmental, and social characteristics in complex interactions, play an important role in determining health status (Jensen et al., 2013). In the context of individual health promotion, the ability to access, understand, assess, and apply health information (Sørensen et al., 2012) underpins both the public health function and social configuration of PL. Sedentary behavior (Zhang et al., 2023) and physical inactivity (Lee and Kim, 2018) may lead to obesity, with more than 34.3% of adults in China being overweight and an obesity prevalence of 16.4%(Pan et al., 2021), which is typically reflected in lower PL scores (Elsborg et al., 2021). Coaches need more attention, as a direct group of PL and social allocation (Cho et al., 2020). Our study validates the phenomenon of coaches perceived PL to advance the further promotion of PL.

The Perceived Physical Literacy Instrument (PPLI), which was first developed by the SUM team in 2016 and tested by physical education teachers (Sum et al., 2018), may be appropriate for sports club coaches. The PPLI was validated by physical education teachers (Sum et al., 2016), adolescents (Sum et al., 2018; Pan et al., 2021), and undergraduate students (Ma et al., 2020). PPLI is widely used in different language versions, such as Cantonese (Sum et al., 2016), Simplified (Traditional) Chinese (Ma et al., 2020; Liu et al., 2022), Spanish (Mendoza-Muñoz et al., 2023), French (Gandrieau et al., 2023), Turkish (Munusturlar and Yıldızer, 2019), and Persian (Samadi et al., 2022). According to Bernstein (1990), PPLI, in the field of knowledge production, may be a reproduction of physical or health education (Young et al., 2020). Our study further validates the phenomenon of knowledge reproduction in sports club coaches through PPLI.

Based on the above data collection and organization, our study focuses on the psychometric properties of the PL structure of sports club coaches through the PPLI (H0), the phenomenon of their knowledge reproduction in the field of PL and public health through the PPLI (H1), and addressing the social configuration of their PL through the PPLI (H2).

## 2 Methods

Our study is a cross-sectional, randomized, observational study. At the time of the study’s inception, the researchers obtained ethical review approval from the University of Work (Document No. NTNC-2024PE-008).

### 2.1 Expert working groups

Two expert working groups were established. The first group, consisting of one professor, two associate professors, and three doctors, helped the authors determine the selection, design, and reliability tests for the PPLI. A second group, consisting of one professor and two associate professors in the English program, helped the authors determine the English–Chinese (Simplified Chinese) translation of the PPLI.

### 2.2 Design of PPLI

All authors, together with the first panel of experts, had a total of two meetings. In the first meeting, they discussed the PL conceptual basis for determining the PPLI. The first version of the PPLI was designed based on Whitehead’s PL concept, and it contained (Chen et al., 2020) entries that may correspond to the six attributes of the PL concept, which are motivation, confidence, physical ability, knowledge, environment, and understanding. Based on the International Physical Literacy Association (IPLA) 2017 PL concept (International Physical Literacy Association, 2007) and Whitehead’s PL concept, for an extrapolated comparison of the concepts, the experts and the authors concluded that the difference is the communicative attribute. Based on the fact that Chinese education uses Bloom’s Taxonomy of Instructional Objectives (University of Illinois Chicago, n.d.), which is similar to Canada’s holistic health and physical education goals, the group of experts and the authors further analyzed the conceptual outreach of PL in Canada. The Canadian conceptualization of PL encompasses the physical, behavioral, emotional, and cognitive domains, and has a correspondence with the IPLA conceptualization of PL (PHE Canada's National Office, n.d.). Relying on Bloom’s Taxonomy of Instructional Objectives (Cognitive, Emotive, and Operational) of Chinese educational practices (Li and Y, 2020), our study suggests that the PPLI may be able to respond to the conceptual outgrowth of PL in Canada. The Emotion domain encompasses motivation and confidence; the Physical domain encompasses physical ability; the Cognitive domain encompasses knowledge and understanding; and the Behavior domain encompasses behavioral participation in lifelong physical activity. The authors and experts agreed that the PL philosophy of sports club coaches may be characterized by a more salient (Canadian PL) psychological profile and by a salient knowledge reproduction profile (Bloom’s pedagogical practices).

For the second meeting, we discussed determining the number of PPLI items. The PPLI was validated by physical education teachers, to obtain a three-factor (sense of self and self-confidence, self-expression and communication with others, and knowledge and understanding), nine-item (items 2, 4, 5, 7, 8, 11, 12, 13 and 17) structural model that detected three attributes of Whitehead’s PL concept, which is item of exploratory factor analysis ranged between 0.69 and 0.87, Cronbach’s α ranged between 0.73 and 0.76, and confirmatory factor analysis (CFA) ranged between 0.48 and 0.83 (Sum et al., 2016). The experts and authors noted that physical education teachers and sports club coaches may differ in relevant characteristics of perceived PL. We therefore used 18 items of PPLI. The PPLI is licensed for use by the SUM team through the open access rights of the research paper, and our research is licensed for use. For the 18 items in the English version of the PPLI, the authors, together with a second team of experts, worked on an English-Chinese (Simplified Chinese) translation, maintaining the linguistic style and the order of the items. A back-translation technique was employed (one author and an English professor translated from English to Simplified Chinese, then another author and an English associate professor translated from Simplified Chinese to English, and a third English associate professor conducted a bidirectional translation check).

To improve the validity of the PPLI, the authors added six additional items to the basic information section (gender, age, sports, coaching level, student, and education). It was not our intention to analyze demographic variables; sociodemographic variables were added only to increase the reliability screening of the sample.

The subjects of our study were club coaches. The inclusion criteria were (Whitehead, 1993) aged 18 or older and (Whitehead, 2001) professionally qualified. The exclusion criteria were (Whitehead, 1993) under 18 years of age and (Whitehead, 2001) not professionally qualified. During the questionnaire design process, we set mandatory fields (basic information items) that had to be filled in before the questionnaire could be submitted (to generate data).

### 2.3 Data collection and sampling

This study is a cross-sectional observational study of a group of sports club coaches. The questionnaire was conducted from August to December 2024.

The sports club coaches were spread across different campuses and clubs, so an anonymous snowballing approach was taken. The questionnaire was created on the Questionnaire Star online platform to generate an electronic QR code for distribution. The questionnaires were reviewed by the College’s Athletic Department Ethics Committee prior to distribution (Document no. NTNC-2024PE-008). The first round of questionnaire distribution was done by the authors (one international referee and national coach, one international coach and national referee) and the first group of experts. After each respondent filled out the survey, they were encouraged to share the QR code with other coaches’ WeChat groups (snowballing approach) to mobilize more participants. Based on the ideal and optimal models (5–10 people per sample item), we set a target of 20 people per sample item, resulting in a minimum total of 360 participants (20 people × 18 items) to be collected (Arbuckle, 2011; Hair et al., 2006; Nunnally and Bernstein, 1967). Decisions on whether to continue, increase, or stop data collection were made accordingly.

The preparation of the questionnaire for reliability and validity included the following entries: (Whitehead, 1993) In the preamble of the questionnaire, the objectives of the study were explained to the respondents and the questionnaire was filled in as a way of obtaining informed consent; (Whitehead, 2001) the respondents were allowed to withdraw or refuse to answer any question at any time; and (Whitehead, 2010) the participants were informed to fill in the questionnaire with anonymous participation and that all the data were for statistical purposes only (there was no personal information to fill in).

### 2.4 Data analysis

The overall data calculation is divided into four stages.

In the first stage, it is about data preparation. The SPSS24 online analysis platform was chosen as the analysis tool (SPSSAU, n.d.). Data precision was retained using rounding criteria to retain percentile decimals (unless specifically requested). The content validity test of the PPLI was conducted, and the experts of the first working group were invited to take charge of it. Experts were invited to assess the content relevance of each item of the PPLI using a four-point Likert scale (1 = not relevant, 4 = highly relevant). Content validity indices (CVI) were calculated, first at the item level (I-CVI) (0.8 or above, as an acceptable criterion) and then at the scale level (S-CVI) (0.9 or above, as an acceptable criterion; Shi et al., 2012). For testing this chance agreement, Fleiss Kappa, which incorporates content validity correction, is an effective method (first group of experts: number 6 > 2, > 0.74, excellent; SPSSAU, n.d.; Shi et al., 2012).

The second stage is data cleaning. Descriptive statistics (percentages and frequencies) were used for eligibility, focusing on the adequacy of the sample (age, etc.). The total sample was tested for internal consistency using Cronbach’s α (>0.6, an acceptable standard; Jöreskog and Sörbom, 1981). The total sample was then randomized sequentially using a computer [according to the two principles of being satisfied with the minimum amount of data and the 1:1 ratio (Tabachnick et al., 2007)], divided into two subsets, and Cronbach’s α values were calculated for each internal consistency test. To ensure the independence and representativeness of each subset, a simple random sampling method was used to represent the universality of the study/sample. The specific process involved using a computer random function to generate a random sequence, which was then matched with the sample ID (Tabachnick et al., 2007). The subset sampling adequacy was then assessed using the Kaiser–Meyer–Olkin (KMO) index (>0.8; Sousa and Rojjanasrirat, 2010).

In the third stage, exploratory factor analysis (EFA) calculations were performed. The first subset was used to determine the factor structure of the PPLI to carry out EFA calculations. To obtain ideal parameter estimates, maximum likelihood estimation was used by Varimax rotation (factor >0.4, otherwise deleted; Shi et al., 2012), preventing situations where the sample size is small or the kurtosis of the variables is unsatisfactory. For the principal component analysis (PCA) of EFA, the correlation of scale items was calculated using the Bartlett’s test of sphericity (*p* ≤ 0.001), the covariance of each item (> 0.40), and the factor loadings (≥ 0.32; Sousa and Rojjanasrirat, 2010).

In the fourth stage, CFA calculations were performed. The second subset was used to refine and characterize the factor structure of the PPLI to carry out CFA calculations. The model fitting practice of CFA, focusing on the structural model, was used to calculate the absolute, parsimonious, and incremental fit indices. Absolute model fitting was performed using the root mean square error of approximation (RMSEA, < 0.1) and the adjusted goodness-of-fit index (AGFI, >0.9). Parsimonious model fitting was performed using the parsimonious normative fit index (PNFI, >0.5), and incremental model fitting was performed using the comparative fit index (CFI, >0.95), the normative fit index (NFI, >0.95), and the Tucker-Lewis index coefficient (TLI, >0.95; Jöreskog and Sörbom, 1981; Bentler and Bonett, 1980; Schreiber et al., 2006).

## 3 Results

### 3.1 Sample characteristics

The data collection time for this study was 25 days, with 486 valid responses to fulfill the sample adequacy requirement (180 for each of the two subsets of the basic objective).

In terms of content validity, there is acceptable content validity, with the I-CVI of the PPLI ranging from 0.8 to 1 and the S-CVI above 0.9 (S-CVI = 0.94). Missing values in the dataset were estimated using the expectation maximization algorithm (α coefficient, α = 0.97, >0.7), indicating that internal consistency was satisfactory and acceptable.

This study was not intended to be a controlled or sequential study, but we performed demographic characterization, conducted an eligibility review, and found that the data were able to meet the basic requirements of this study (Table 1).

**Table 1:** Demographic characteristics of the club coach (*n* = 486).

Total N	*N* = 486	%
Gender		
Men	214	44.03
Women^*^	272	55.97
Age		
18–29^*^	189	38.89
30–39	174	35.80
40 and above	123	25.31
Sport		
Physical shape	151	31.07
Physical fitness^*^	184	37.86
Other	151	31.07
Coaching Levels		
Junior	169	34.77
Intermediate^*^	229	47.12
Senior	88	18.11
Students		
Young children	150	30.86
Adolescents^*^	266	54.74
Adults	70	14.40
Education		
Specialties	146	30.04
Undergraduates^*^	262	53.91
Masters	54	11.11
Doctors	24	4.94

*, predominating.

(1) Gender: 44.03% were men (214) and 55.97% were women (272), with a surplus of women over men. (2) Age: 18–29 years (189) accounted for 38.89%, 30–39 years (174) accounted for 35.8%, and 40 years and older (123) accounted for 25.31%, with the 18–29-year-old age group predominating. (3) Sports: sports for shape 31.07% or 151, sports for fitness 37.86% or 184, other sports 31.07% or 151, and about average in each category. (4) Levels: junior 169 or 34.77%, intermediate 229 or 47.12%, senior 88 or 18.11%, with intermediate levels predominating (probably age-related, in line with the age profile). (5). Students: 30.86% of young children 150, 54.74% of adolescents 266, and 14.40% of adults 70, with adolescents predominating (in line with the situation in China). (6). Education: 146 or 30.0% for specialties, 262 or 53.91% for undergraduates, 54 or 11.11% for masters, and 24 or 4.94% for doctors, with undergraduates predominating (in line with China’s education situation).

### 3.2 Characteristics of the dataset

Contesting the 486 valid data, two subsets (243 each) were obtained which based on computer production of random sequences (1:1 ratio). The first subset, used for EFA (Cronbach’s α), had an α coefficient of 0.97 (>0.7), which is acceptable for internal consistency. The second subset, used for CFA (Cronbach’s α), had an α coefficient of 0.97 (>0.7), which is acceptable for internal consistency. See Table 2.

**Table 2:** Content validity of the data.

Subset	*N* = 486	Used	Cronbach α
First	243	EFA	0.97 (>0.7)
Second	243	CFA	0.97 (>0.7)

### 3.3 Exploratory factor analysis (EFA)

Principal component analysis (PCA calculations performed for the EFA resulted in a final nine-item scale of 18 items for the PPLI (*n* = 243) with four factors, as shown in the pattern matrix in Table 3.

**Table 3:** Factor structures by exploratory factor analysis and reliability.

Sign	F1	F2	F3	F4	CITC	Communality (h^2^)	Scale α
PL09	0.70	0.29	0.43	0.23	0.80	0.81	0.87
PL15	0.70	0.37	0.23	0.33	0.76	0.79	
PL17	0.75	0.26	0.29	0.34	0.77	0.82	
PL10	0.32	0.75	0.24	0.37	0.75	0.86	0.83
PL11	0.33	0.75	0.36	0.22	0.74	0.86	
PL07	0.37	0.27	0.75	0.28	0.78	0.85	0.83
PL03	0.29	0.34	0.72	0.35	0.78	0.84	
PL01	0.29	0.37	0.33	0.72	0.84	0.85	0.83
PL06	0.40	0.25	0.30	0.73	0.79	0.86	
PE	2.23	1.80	1.79	1.72			
%OV	24.77	20.05	19.91	19.11			
C%	24.77	44.82	64.72	83.83			

CITC, Corrected Item-total Correlation; PE, Present Eigenvalues; %OV, % of Variance; C%, Cumulative %.

The four-factor model loaded nine items, explaining 83.83% of the variance, including 1, 3, 6, 7, 9, 10, 11, 15, and 17. Factor correlation validation indicated adequate relevance among structural factors. Factor loadings for the nine items ranged from 0.70 to 0.75 (>0.32). The total correlations for the calibration items ranged from 0.79 to 0.86 (>0.4). Item content consistency results were met with α of 0.87, 0.83, 0.83, and 0.83 (>0.7) for the four factor scales. The result of the validation sample adequacy was found to be largely satisfactory with a KMO index of 0.96 (>0.8). The *p*-value of the Bartlett’s test of sphericity is 0.000 (*p* ≤ 0.001), indicating that the validation sample scale correlation results as suitable for PCA. The first removal of the 11 cross terms (Whitehead, 1993; Whitehead, 2001; Whitehead, 2010; Whitehead, 2019; Bailey et al., 2023; Coaching Ireland, 2024; Gandrieau et al., 2023; Lundvall, 2015; Robinson and Randall, 2017; Caldwell et al., 2021; Chen et al., 2020) was justified on the grounds that they loaded 0.40 or higher on two or more factors. However, further PCA analyses of the EFA yielded unsatisfactory results due to the presence of single-topic items. After the addition of items 1 and 3, it gave a four-factor (with cross items, but with higher factor loading). EFA test of the four factors: present eigenvalues 1.72–2.23, % of variance 19.11–24.77%, and % of cumulative 24.77–83.83%.

### 3.4 Confirmatory factor analysis

**Figure 1:**
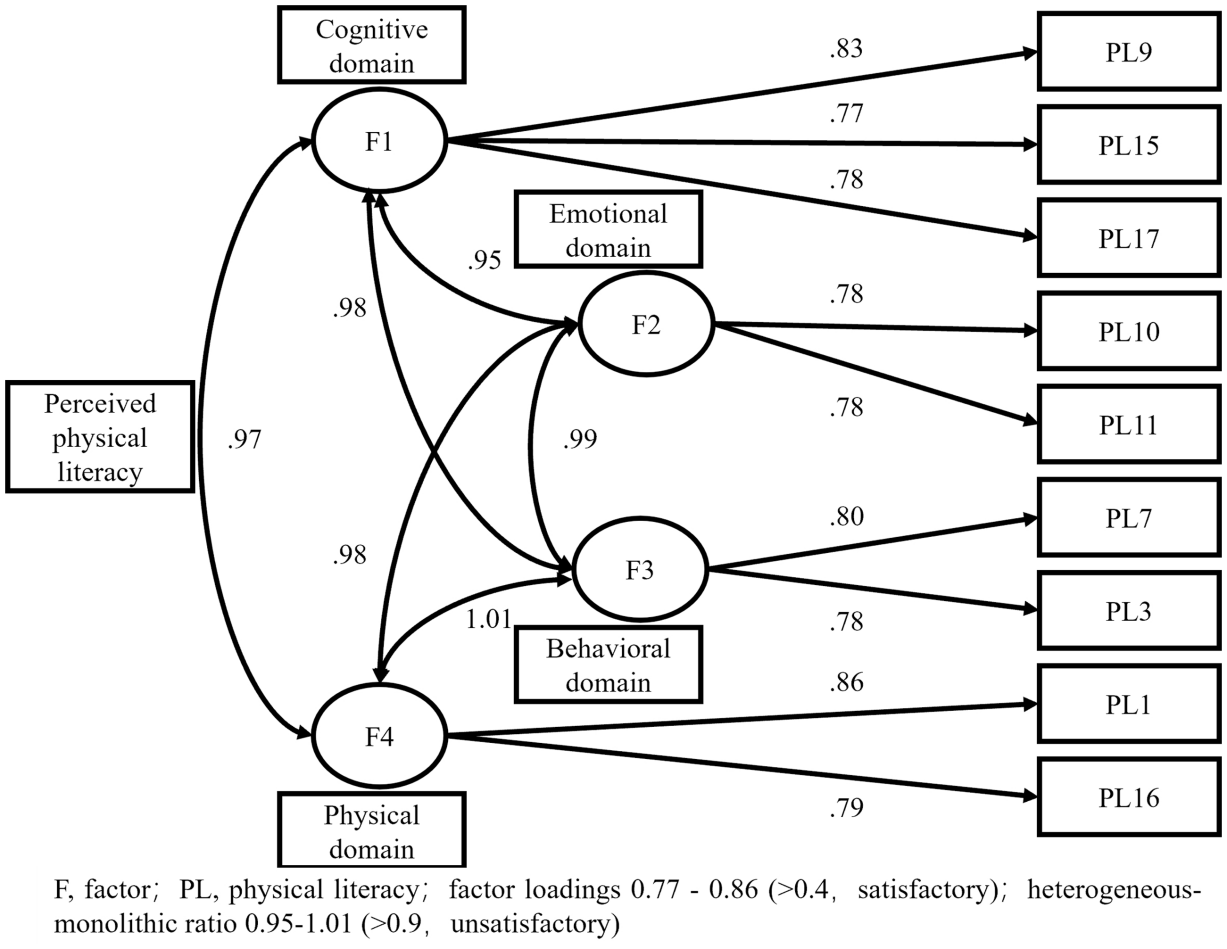
Factor structure and standardized factor loading on perceived physical literacy items.

Cross-validation was performed through CFA, and nine PPLIs were retained, confirmed to be acceptable four-dimensional structural models (Figure 1).

The CFA’s factorial validity results were satisfactory, with factor loadings ranging from 0.77 to 0.86 (>0.4) for the nine instruments. The structural model fit for CFA (n = 243) was high with a standardized chi-square index of 2.40 (<3.00). The absolute model fit index RMSEA was 0.08 (<0.10) and AGFI was 0.91 (>0.90). The incremental fit of the model was strong with an NFI of 0.97, CFI of 0.98, and TLI of 0.97 (all > 0.95). The parsimonious fit of the model was acceptable with a PNFI of 0.56 (>0.5). See Table 4.

**Table 4:** Factor structures by confirmatory factor analysis and reliability.

(A). Factor loading coefficient table							
F/D	IN	FL	SE	CR	P	STD	R^2^
F1	PL9	1.00	-	-	-	0.83	0.79
F1	PL17	0.91	0.07	13.79	0.00	0.77	
F1	PL15	0.91	0.07	13.85	0.00	0.78	
F2	PL10	1.00	-	-	-	0.78	0.78
F2	PL11	1.08	0.08	12.88	0.00	0.78	
F3	PL7	1.00	-	-	-	0.80	0.79
F3	PL3	0.91	0.07	13.72	0.00	0.78	
F4	PL1	1.00	-	-	-	0.86	0.83
F4	PL6	0.84	0.06	15.21	0.00	0.79	
IN, item number; F/D, factor/domain; FL, factor loading; SE, standard error; CR, critical ratio; P, p-value; STD, standardized factor loading; R2, squared multiple correlation; −, Reference items.							

(B). Cross-validation by confirmatory factor analysis and reliability.						
Index	*χ*^2^/df	RMSEA	NFI	CFI	TLI	PNFI
Standards	<3.00	<0.10	> 0.95	> 0.95	> 0.95	> 0.50
Value	2.40	0.08	0.97	0.98	0.97	0.56
*χ*^2^/df, standardized chi-square index; RMSEA, root mean square error of approximation; NFI, normative fit index; CFI, comparative fit index; TLI, Tucker-Lewis index; PNFI, parsimonious normative fit index.						

A notable indicator is the heterogeneous-monolithic ratio 0.95–1.01 (>0.9, unsatisfactory). This indicates that the clarity between the four dimensions of the structural model is not high. See Figure 1.

## 4 Discussion

The results of the data showed that the four factors of the structure of the PPLI of the sports club coaches, with good validity, answered the characteristics of the psychological structure of their PL (H0). From the data collected, the PPLI has been validated several times for different occupations and ages, and each validation has achieved good validity (Cho et al., 2020; Sum et al., 2018; Pan et al., 2021; Ma et al., 2020; Liu et al., 2022; Mendoza-Muñoz et al., 2023; Munusturlar and Yıldızer, 2019; Samadi et al., 2022). Our study, focusing on the differences from the audience’s perspective, further extends the applicability of the PPLI.

### 4.1 The phenomenon of “knowledge reproduction”

The PPLI structural model of sports club coaches has four dimensions, including the first cognitive dimension, the second emotional dimension, the third behavioral dimension, and the fourth physical dimension (Table 5).

**Table 5:** Items of the versions of perceived physical literacy instrument.

Items	Descriptions	Physical teachers	Sports club coaches
PL01	I possess adequate fundamental movement skills.		F ^b^ 4
PL02	I am physically fit, in accordance to my age.	F ^a^ 3	
PL03	I am able to apply learnt motor skills to other physical activities.		F ^b^ 3
PL04	I have a positive attitude and interest in sports.	F ^a^ 1	
PL05	I appreciate myself or others doing sports.	F ^a^ 1	
PL06	I am able to apply PE knowledge in the long run.		F ^b^ 4
PL07	I possess self-management skills for fitness.	F ^a^ 3	F ^b^ 3
PL08	I possess self-evaluation skills for health.	F ^a^ 3	
PL09	I am willing to do sports for better health.		F ^b^ 1
PL10	I have strong communication skills.		F ^b^ 2
PL11	I have strong social skills.	F ^a^ 2	F ^b^ 2
PL12 ^a^	I am confident in wild/natural survival.	F2	
PL13	I am capable in handling problems and difficulties.	F ^a^ 2	
PL14	I have a mindset for lifelong sports.		
PL15	I can turn doing sports into an ongoing habit of life.		F ^b^ 1
PL16	I establish friendship through sports.		
PL17	I am aware of the benefits of sports related to health.	F ^a^ 1	F ^b^ 1
PL18	I aspire to know the current sports trend.		

F ^a^ 1, Knowledge and Understanding; F ^a^ 2, Self-expression and Communication with Others; F ^a^ 3, Self-awareness and Confidence; F ^b^ 1, Cognitive Dimension; F ^b^ 2, Emotional Dimension; F ^b^ 3, Behavioral Dimension; F ^b^ 4, Physical Dimension.

The first dimension consists of items 9, 15, and 17. Item 9 corresponds to the question, “I am willing to do sports for better health.” Item 15 corresponds to the question, “I can turn doing sports into an ongoing habit of life.” Item 17 corresponds to the question, “I am aware of the benefits of sports related to health.” According to Whitehead’s view of PL (Whitehead, 1993; Whitehead, 2001; Whitehead, 2010; Whitehead, 2019), this corresponds to knowledge and understanding for its external core, and according to the Canadian view of PL (ParticipACTION, 2025), this corresponds to the cognitive domain.

The second dimension consists of items 10 and 11. Item 10 corresponds to the question, “I have strong communication skills.” Item 11 corresponds to the question, “I have strong social skills.” According to Whitehead’s view of PL (Whitehead, 1993; Whitehead, 2001; Whitehead, 2010; Whitehead, 2019), this corresponds to self-expression and communication with others for its external core, and, according to the Canadian view of PL (ParticipACTION, 2025), this corresponds to the emotional domain.

The third dimension consists of items 7 and 3. Item 7 corresponds to the question, “I possess self-management skills for fitness.” Item 3 corresponds to the question, “I am able to apply learnt motor skills to other physical activities.” According to Whitehead’s view of PL (Whitehead, 1993; Whitehead, 2001; Whitehead, 2010; Whitehead, 2019), this corresponds to a sense of physical self and self-confidence for its external core. According to the Canadian view of PL (ParticipACTION, 2025), this corresponds to the behavioral domain.

The fourth dimension consists of items 1 and 6. Item 1 corresponds to the question, “I possess adequate fundamental movement skills.” Item 6 corresponds to the question, “I am able to apply PE knowledge in the long run.” According to Whitehead’s view of PL (Whitehead, 1993; Whitehead, 2001; Whitehead, 2010; Whitehead, 2019), this corresponds to the sense of physical self and self-confidence for its kernel core, and, according to the Canadian view of PL (ParticipACTION, 2025), this corresponds to the physical domain.

In contrast to physical education teachers, our study monitored all the external and some of the kernel of Whitehead’s conceptualization of PL (Sum et al., 2016; Sum et al., 2018). Whether the observations, because of the physical domain, are the cause of the occupational characteristics of sports club coaches, we have not been provided with direct evidence. However, the phenomenon of “assessment with knowledge reproduction,” according to Bernstein (1990), has been verified in the observation of PL among sports club coaches (H1).

An interesting data indicator is the heterogeneous–monolithic ratio 0.95–1.01 (>0.9, unsatisfactory). This indicates that the clarity between the four dimensions of the coaches’ structural model is not high, and a clearer expression is that the coaches’ perception of PL characteristics is more general/vague. This differs from physical education teachers (Sum et al., 2016), who place greater emphasis on teaching objectives (distinguishing concepts, which may lead to better teaching outcomes). Coaches, on the other hand, place greater emphasis on students’ competition results (the more direct and simpler, the better the training or competition outcomes may be). The heterogeneous–monolithic ratio metric indicates that the knowledge reproduction characteristics of physical education teachers and coaches differ.

### 4.2 Social configuration

It is very interesting to note that the factor loadings for the 9 items (Whitehead, 2001; Whitehead, 2019; Bailey et al., 2023; Coaching Ireland, 2024; Gandrieau et al., 2023; Lundvall, 2015; Robinson and Randall, 2017; Caldwell et al., 2021; Chen et al., 2020) that we deleted as cross-items were all above 0.4, and their Cronbach’s α coefficient was 0.95. Our attempts to explore more items placed in all four dimensions failed. We also explored that items 5 and 13 could be used as a dimension. Item 5 corresponds to the question, “I appreciate myself or others playing sports.” Item 13 corresponds to the question, “I appreciate myself or others playing sports.” According to Whitehead’s view of PL (Whitehead, 1993; Whitehead, 2001; Whitehead, 2010; Whitehead, 2019; Sum et al., 2018), this corresponds to a sense of interaction with the environment for its kernel core. This may also be a sub-dimension of the behavioral dimension of IPLA (International Physical Literacy Association, 2007), but it was deleted during the attempt to merge it with PL1 and PL6 due to cross-phenomena. A portion of the remaining items is partially reflective of the kernel motivational properties of PL and may be the result of data contingencies (as cross-cutting items).

The three-dimensional, nine-item structural model for physical education teachers (Sum et al., 2016) has the following characteristics: the first dimension, knowledge and understanding (PL 4, 5, and 17), has cognitive characteristics; the second dimension, self-expression and communication with others (PL 11, 12, and 13), has emotional characteristics; and the third dimension, self-awareness and self-confidence (PL 2, 7, and 8), has behavioral characteristics. It is not our intention to compare physical education teachers, but coaches have a relatively comprehensive social configuration in the public health domain, with cognitive (PL9,15,17), emotional (PL10,11), physical (PL3,7), and behavioral (PL1,6) validated (H2), even if items 5 and 13 are deleted. From Bloom’s view of instructional goals (University of Illinois Chicago, n.d.), sports club coaches have relatively comprehensive cognitive, emotional, and operational (physical and behavioral) domains of PL.

### 4.3 Limitations, strengths, and future directions

#### 4.3.1 Limitations

There are several limitations to this paper. First, the questionnaire data were collected using snowball sampling, which may not fully represent the entire population (all age groups were included, but the data were not equally distributed). Second, the aspects of Whitehead’s PL views that were not explored do not mean that sports club coaches do not have relevant PL concepts. Third, the social configuration of PL among sports club coaches requires further correlational research, including factors such as self-identity.

#### 4.3.2 Strengths

Our research validated a nine-item, four-factor structural model. Compared with the nine-item, three-factor structural model of physical education teachers, there are certain differences (multidimensional; Table 5). The coaches’ structural model exhibits more comprehensive structural characteristics, meaning that we observed the entire external core and part of the internal core of Whitehead’s PL concept. The interaction between the internal and external cores of the PL concept in the coaches’ structural model may suggest that PL serves as a powerful empirical basis for addressing public health issues (lack of physical activity and sedentary behavior).

#### 4.3.3 Future directions

Our study has boldly explored the social allocation characteristics of coaches, which may be related to PL. In the future, more correlational and structured verifications are needed, such as hierarchical analyses of coaches’ PL based on sociological characteristics.

## 5 Conclusion

As an instrument for measuring perceived PL, the PPLI has proven to be a reliable and valid instrument for measuring perceived PL among sports club coaches. Based on the results of the EFA and CFA, our study observed that these sports club coaches possess a relatively comprehensive phenomenon of perceived PL. The availability of items, compared to physical education teachers, is a phenomenon that Bernstein describes as “assessment with knowledge reproduction.” The absence of items may be a phenomenon of coaches’ PL being socially misconfigured in the public health domain.

## Data Availability

The original contributions presented in the study are included in the article/supplementary material, further inquiries can be directed to the corresponding author.
